# Burden of Disease Due to Consumption of Alcohol and Other Drugs in Colombia, 2016–2022: A Subnational Regional Analysis

**DOI:** 10.3390/ijerph23050659

**Published:** 2026-05-15

**Authors:** Oscar Alexander Gutiérrez-Lesmes, Emilce Salamanca Ramos, Karen Julieth Quintero Díaz

**Affiliations:** 1 School of Public Health, Universidad de los Llanos, Villavicencio 500001, Meta, Colombia; oagutierrez@unillanos.edu.co; 2 Gesi Research Group, School of Public Health, Universidad de los Llanos, Villavicencio 500001, Meta, Colombia; esalamanca@unillanos.edu.co

**Keywords:** disability-adjusted life years, consumption of alcohol, substance-related disorders, Colombia

## Abstract

**Highlights:**

**Public health relevance—How does this work relate to a public health issue?**

- Alcohol and psychoactive substance use generated 236,154.42 DALYs in Colombia between 2016 and 2022.
- The burden associated with alcohol and psychoactive substance use increased across Colombian departments during the study period.

**Public health significance—Why is this work of significance to public health?**

- This study estimated the burden of disease attributable to alcohol and psychoactive substance use in Colombia using DALYs and WHO Global Health Estimates methods.
- Important territorial differences were identified, with the highest DALY rates observed in Quindío, Nariño, and Norte de Santander.

**Public health implications—What are the key implications or messages for practitioners, policy makers and/or researchers in public health?**

- The findings support the need to identify the most affected territories and prioritize allocation of health resources according to regional needs.
- The study provides a baseline to monitor interventions and public policies related to alcohol and psychoactive substance use in Colombia.

**Abstract:**

Alcohol and psychoactive substance use represent a major burden for global public health, increasing the risk of non-communicable diseases, violence, road traffic injuries, dependence, and mental disorders, and generating impacts on productivity and social welfare. This study aimed to estimate the burden of disease attributable to alcohol and other psychoactive substances in the departments of Colombia from 2016 to 2022. A burden-of-disease study was conducted using the Disability-Adjusted Life Years (DALYs) indicator, following the methodology of the World Health Organization Global Health Estimates. Official morbidity and mortality databases were used. An estimated 236,154.42 DALYs were attributable to alcohol and psychoactive substance use in Colombia during the study period, increasing from 14,158.7 DALYs in 2016 to 40,190.7 DALYs in 2022. The burden was heterogeneous across departments, with values above 1000 DALYs in Quindío (1779.5), Nariño (1624.3), and Norte de Santander (1008.0) and below 132 DALYs in La Guajira, Casanare, and Vaupés. Men accounted for 73.5% of total DALYs. The mean age of morbidity records associated with alcohol and psychoactive substance use disorders was 30.67 years in men and 32.37 years in women. The burden associated with psychoactive substance use is increasing in Colombia, with differences by sex and department of residence.

## 1. Introduction

Consumption of alcohol and psychoactive substances represents one of the most significant burdens for global public health due to its impact on premature mortality, disability, and multiple social and economic consequences [1,2]. Their use is associated with a greater risk of non-communicable diseases, external injuries, violence, mental disorders, and dependency, generating a sustained impact on productivity and social welfare [3,4,5,6].

According to the 2016 Global Burden of Disease (GBD) study, alcohol consumption was responsible for approximately 132.6 million Disability-Adjusted Life Years (DALY), equivalent to 5.1% of the global burden of disease and injury, as well as nearly 2 million premature deaths during that same year [7,8]. In comparison, consumption of illicit drugs caused around 31.8 million DALYs (1.3% of the global burden), although alcohol accounted for close to 76% of the total burden attributable to the use of psychoactive substances [9]. These findings consolidate alcohol as one of the most relevant risk factors globally, occupying the seventh place among all the risk factors and the first place in the age group from 15 to 49 years [7].

The burden associated with alcohol and drug consumption is particularly high in young populations; the 2019 GBD estimated that, in individuals from 10 to 24 years, alcohol was responsible for 5.9 million DALYs and drug consumption was responsible for 4.1 million DALYs with a higher impact on men [9].

In South America, regional analyses based on the 2019 GBD documented that DALYs due to disorders related to cocaine and cannabis have diminished since 2010; however, higher rates prevail in men [10,11]. Within this context, Colombia had an incidence of disorders due to cannabis consumption and a burden due to disability greater than the regional averages [11].

Nationally, it was estimated that in Colombia nearly 7563 deaths were attributable to alcohol in 2019, of which approximately 50% corresponded to injuries from external causes [12]. Colombia is administratively divided into 32 departments and one capital district (Bogotá, D.C.), which constitute the country’s first subnational administrative level. Despite the magnitude of the problem, no systematic DALY calculations exist attributable to the consumption of alcohol and other drugs at the departmental (first-level administrative divisions equivalent to states or provinces) levels through standardized methodologies, such as those by the GBD or the Global Health Estimates (GHE).

Subnational information available is limited to the National Survey of Psychoactive Substance Consumption (ENCSPA), which provides prevalence rates by department [13]. Nevertheless, the lack of territorial estimations of the burden of disease limits the capacity to identify regional inequalities, prioritize interventions, and guide the allocation of resources in public health.

Despite the growing epidemiological and public health relevance of alcohol and psychoactive substance use in Colombia, the available evidence remains largely limited to prevalence surveys and national-level estimates. Subnational analyses of the burden of disease are still scarce, particularly those based on standardized Disability-Adjusted Life Year (DALY) methodologies. This lack of territorial estimates restricts the ability to identify regional inequalities, characterize geographic patterns of disease burden, and guide the allocation of health resources and interventions according to local epidemiological needs.

Within this context, conducting a study on the burden of disease attributable to the consumption of alcohol and other drugs in Colombia and its departments is essential to assess its impact on public health. Therefore, the objective of this study was to estimate the burden of disease attributable to the consumption of alcohol and other drugs in Colombia and its departments during the period from 2016 to 2022. This analysis permits identifying the most affected territories, prioritizing the allocation of resources in clinical services adapted to regional needs, and establishing a baseline to monitor and assess interventions and public policies, contributing to strengthening local evidence in the face of gaps in global studies, which often fail to capture the country’s critical heterogeneities.

## 2. Materials and Methods

The study of the burden of disease due to the consumption of alcohol and other psychoactive substances was conducted in the departments of Colombia during the period from 2016 to 2022, using the methodological guidelines used by the WHO in GHE studies 2024 [14].

Secondary data from publicly accessible national health information systems were used in this study, including SISPRO, RIPS, and RUAF. SISPRO is Colombia’s official national health information system and integrates nationwide administrative health databases with broad population coverage. The RIPS database contains mandatory records of healthcare services delivered by public and private healthcare providers across the country, whereas RUAF compiles national mortality records, including deaths occurring both within and outside healthcare facilities. These systems are routinely used for epidemiological surveillance, health services monitoring, and population health research in Colombia. To avoid duplicate records, morbidity cases were identified and filtered using unique individual identification numbers available in SISPRO databases. Therefore, each morbidity case included in the analyses represented a unique individual rather than repeated healthcare encounters.

To estimate morbidity associated with alcohol and psychoactive substance use disorders, medical care records from the hospital care system were used, classifying cases according to the principal diagnosis recorded in the RIPS database and for mortality data, death certificates from the records of forensic medicine and the hospital care system were used, selecting cases by underlying cause of death, stored in the RUAF database. For the denominators of the rates, population projections by department, year, and sex by the Colombian National Department of Statistics (DANE) were used.

All the cases registered in the databases consulted for the study period with a diagnosis within the group of alcohol and other drug use disorders were included. The study estimated the burden directly associated with alcohol and psychoactive substance use disorders identified through ICD-10 diagnostic codes recorded as principal diagnoses or underlying causes of death, rather than the full attributable burden of all diseases causally linked to substance use. Therefore, the estimates should be interpreted as a conservative approximation of the total burden attributable to alcohol and psychoactive substance use in Colombia.

Cases classified in codes ICD-10: F190-199, X420-429, and X450-459, were distributed proportionally according to age group, sex, and department to assign them to the different substances presented in the research.

Data downloaded from SISPRO are organized in pivot tables (Supplementary Materials Table S1). This information was processed in Power BI Desktop and Power Query to construct individual record databases. Once the mortality and morbidity databases were adjusted, calculation of years of life lost (YLL) due to premature mortality, years lived with disability (YLDs), and disability-adjusted life years (DALY) was performed using the WHO’s simplified GHE formulas [15].

The DALY indicator combines premature mortality and disability into a single summary measure of population health loss. It is composed of years of life lost (YLLs) and years lived with disability (YLDs). The YLL refers to the years a person stops living when they die before reaching a theoretical life expectancy. YLDs quantify the loss of healthy life associated with disability and non-fatal health states. The calculation expression is as follows:

Equation (1): Basic formula for DALYs.

(1) $${DALY}_{c,s,a,t}={YLD}_{c,a,s,t}+{YLL}_{c,a,s,t}$$

YLDs are years lived with disability; YLL are years of life lost; consumption of alcohol and other substances (*c*), age (*a*), sex (*s*), and year of occurrence (*t*). 

To calculate years of life lost, the number of cases of mortality whose recorded underlying cause was consumption of alcohol and other substances was used, according to department, age, and sex, and the calculation was made by applying the following formula:

Equation (2): Basic formula for YLLs.

(2) $${YLL}_{c,a,s,t}=D_{c,a,s,t}e_{x}^{*}$$

(*D*) is the number of deaths due to consumption of alcohol and other substances (*c*), age of occurrence of death (*a*), sex (*s*), and year of occurrence (*t*) and e_x_* is the life expectancy at each age (weighting factor derived from the standard life expectancy recommended by the WHO). 

To calculate the years of life lived with disability, the prevalence of consumption of alcohol and other substances was estimated, according to the principal diagnosis reported in the RIPS by applying the following formula:

Equation (3): Basic formula for YLDs.

(3) $${YLD}_{c,a,s,t}=W_{c}*P_{c,a,s,t}$$

(*W*) is the weight of disability and (*P*) the prevalence of consumption of alcohol and other substances (*c*), age group (*a*), according to sex (*s*), in year (*t*)*,* using disability weights from the 2019 GBD for each of the health states.

The mathematical calculations of YLLs, YLDs, DALYs, and uncertainty intervals were performed using spreadsheet-based calculation tools and IBM SPSS Statistics™ version 23 software, licensed by Universidad de los Llanos, on HP equipment manufactured in Palo Alto, California, United StatesPotential sources of bias included underreporting, limited access to healthcare services, and misclassification of causes of death. Mitigation strategies included the incorporation of forensic medicine mortality records and the use of standardized WHO burden-of-disease methods.

Ethical considerations: This research was categorized as minimal risk; requirements by the GATHER guide for transparent presentation of population health estimates were applied (see Supplementary Materials Table S2). In addition, it complied with the ethical principles of Resolution 8430 of 1993 [16] for health research in Colombia and the CIOMS guides [17].

The data comes from secondary sources of information, collected by state entities with objectives framed within national policies on health care and surveillance, with collection regulated by norms that include mandatory registry of this information by government authorities. The said information is of a public nature and freely accessible. The databases used do not contain information that allows direct or indirect identification of the subjects, and the analysis was performed on aggregated data by spatial unit.

Consequently, no informed consent was needed, according to the CIOMS guideline 12 [17], which defines that informed consent is not necessary when data are collected within the context of routine clinical care and are mandatory to record, population-based, and anonymized.

## 3. Results

During the period from 2016 to 2022, Colombia registered 299,591 cases of morbidity due to the consumption of alcohol and other psychoactive substances. The largest proportion of cases corresponded to the consumption of cannabis, representing 27.9% of the total, followed by the group of other drugs with 22.2% of the cases, and alcohol consumption with 21.7%. Regarding mortality, 1424 mortality cases were registered attributable to the consumption of alcohol and other substances, of which 39.3% correspond to alcohol consumption, followed by the category of other drugs with 31.5% and opioids with 28.9%. (Supplementary Materials Table S3).

With respect to age, differences were found by sex for morbidity and mortality; while in men the mean age for morbidity was 30.90 years (95% UI 30.86–30.99), in women it was 32.90 years (95% UI 32.76–33.02). For mortality, the mean age in men was 42.74 years (95% UI 41.80–43.68), while in women it was 38.36 years (95% UI 35.67–41.06).

When analyzing age by type of substance, cannabis consumption had the lowest mean age, with 23.01 (95% UI 22.93–23.09) and 24.60 (95% UI 24.40–24.82) years for men and women, respectively. In contrast, for mortality, opioids registered the lowest mean age with 34.40 (95% UI 33.13–35.70) years in men and 31.40 (95% UI 31.30–31.50).

The annual and periodic mortality and morbidity cases that occurred in the departments of Colombia can be consulted in (Supplementary Materials Table S3).

### 3.1. Disability-Adjusted Life Years

From 2016 to 2022, inhabitants across the departments of Colombia lost 236,154.42 (95% UI 231,988.29–240,320.55) years of healthy life (DALYs) attributable to the consumption of alcohol and other psychoactive substances as a result of living in suboptimal health conditions and premature mortality, corresponding to a rate of 478.10 (95% UI 469.70–486.50) DALYs per 100,000 inhabitants. During the study period, DALYs in Colombia showed an increasing trend between 2016 and 2022, with an average annual increase of 3261 DALYs, rising from 14,158.70 in 2016 to 40,190.70 DALYs in 2022. Specifically, DALYs increased across all departments.

Classification of the departments according to DALY rates per 100,000 inhabitants evidences territorial differences during the period analyzed, with rates ranging from 27.50 DALYs to 1946.60. The departments with the highest DALY rates are: Quindío 1779.50, Nariño 1624.30, Norte de Santander 1008, Risaralda 909.50, Antioquia 889.20, and Caldas 770.90, as shown in Table 1.

Risaralda, Caldas, and Antioquia share geographic boundaries, while Norte de Santander and Nariño share international boundaries with Venezuela and Ecuador, respectively. In contrast, the departments from the Orinoquia region (Arauca, Casanare, Meta, and Vichada) had the lowest DALY rates during this period.

The annual, period and gender-specific behavior of each department’s YLD, YLL, and DALY rates by year, sex, and department can be found in Supplementary Materials Table S4.

With respect to sex, 73.5% of the country’s DALYs were contributed by men. Men presented a DALY rate of 823.5 (95% UI 807.50–839.50), and women 148.40 (95% UI 142.30–154.50) per 100.000 inhabitants, evidencing greater impact on the male population.

At the departmental level, the highest DALY rate in men and women was recorded in Quindío with 2963.70 (95% UI 2776.30–3151) and 679.40 (95% UI 604.70–754.10), respectively.

The substances contributing most to the country’s DALYs were cannabis (27.6%), followed by other drugs (22.2%) and alcohol (21.8%). It is important to mention that, in the category of other drugs, cases have been recorded involving the combination or use of multiple drugs, including alcohol and other substances used simultaneously.

Differences by substance type also revealed distinct epidemiological patterns. Cannabis-related disorders were associated with younger age groups, whereas alcohol-related burden showed a higher contribution to premature mortality and older age groups [9]. Opioid-related mortality presented lower mean ages, suggesting higher lethality among younger populations [7]. These findings are consistent with international evidence showing heterogeneous health impacts according to substance type, consumption patterns, and associated social contexts [11]. In addition, regional variations in drug-related burden may reflect differences in local drug markets, trafficking routes, and access to health and addiction treatment services [13].

**Figure 1:**
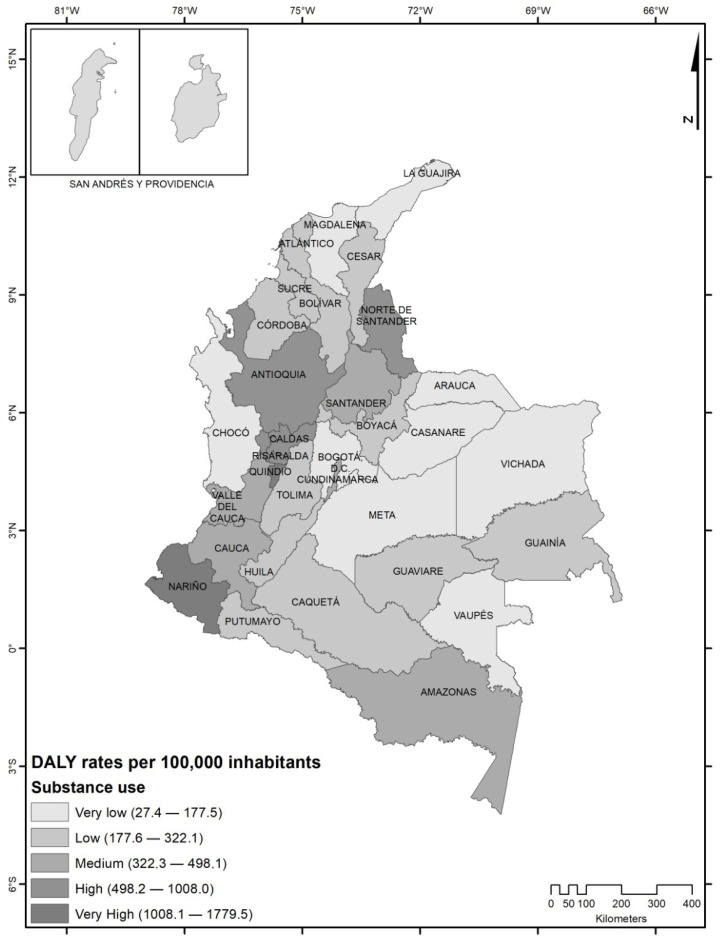
Geographic distribution of disability-adjusted life year (DALY) rates associated with alcohol and psychoactive substance use across Colombian departments, 2016–2022.

The spatial distribution of DALY rates revealed marked territorial heterogeneity across Colombian departments (Figure 1). The highest burden was concentrated in departments located in the Andean and southwestern regions, particularly Quindío, Nariño, Risaralda, Antioquia, and Norte de Santander, which presented high and very high DALY rates. A geographic clustering pattern was observed in the Colombian Coffee-Growing Region (Eje Cafetero), where Quindío, Risaralda, Caldas, and Antioquia showed consistently elevated rates.

Additionally, departments with international borders, such as Nariño and Norte de Santander, also exhibited high DALY burdens. In contrast, departments from the Orinoquía and Amazon regions, including Vichada, Arauca, Casanare, Vaupés, and Guaviare, presented the lowest DALY rates during the study period.

Overall, the spatial distribution suggests substantial geographic inequalities in the burden associated with alcohol and psychoactive substance use in Colombia.

### 3.2. Years of Life Lost (YLLs)

In the departments of Colombia, during the study period, 71,576.30 (95% UI 70,341.66–72,810.94) YLL were lost, with a YLL rate of 144.90 (95% UI 142.40–147.40) per 100,000 inhabitants. Of these, 86.7% were contributed by men. When broken down by type of psychoactive substance, alcohol was the substance that contributed the most to the YLL, with 39.3% of the cases, followed by consumption of other drugs with 31.5% and opioids with 28.9%.

The departments with the highest rates were: Risaralda 289.50 (95% UI 272.10–306.90), Valle del Cauca 279.40 (95% UI 270–288.80), Antioquia 256.20 (95% UI 247.90–264.50), Quindío 228.60 (95% UI 195.50–261.80), and Cauca 219.30 (95% UI 201.50–237.20); the YLL rates for all the departments can be consulted in (Supplementary Materials Table S3). At the national level, a sustained increase in YLL was found during the period studied, with an average increase of 1439 YLL per year, going from 4890.2 YLL in 2016 to 13,218.5 YLL in 2022.

### 3.3. Years Lived with Disability (YLDs)

In all, 164,578.12 YLDs were recorded due to consumption of alcohol and other psychoactive substances (95% UI 163,035.50–166,120.73), with a rate of 333.20 (95% UI 330.10–336.30) YLDs per 100,000 inhabitants; 73.5% of the YLDs were contributed by men.

Throughout the study period, YLDs showed an increasing temporal trend, with an estimated annual increase of 1882.2 YLDs, going from 9.268 in 2016 to 26,891 in 2022. Cannabis was the substance that generated the greatest YLDs (27.8%), followed by other drugs (22.2%) and alcohol with 21.7% of all the YLDs in Colombia during the period analyzed. Supplementary Materials Table S4.

The departments with the highest rates were: Quindío 1550.90 (95% UI 1538.30–1563.50), Nariño 1386.90 (95% UI 1321.60–1452.30), Norte de Santander 831.20 (95% UI 807–855.50), Caldas 642.80 (95% UI 606.50–679.10), and Antioquia 633 (95% UI 621.10–645); the YLDs rate of the remaining departments can be consulted in (Supplementary Materials Table S4).

## 4. Discussion

The results of this study show that consumption of alcohol and other psychoactive substances generates a substantial and growing burden of disease in Colombia, with an estimated loss of 236,154 DALYs between 2016 and 2022, an average annual increase of 3261 DALYs. These findings confirm that substance consumption constitutes a priority public health problem, with sustained implications for premature mortality, disability, and social productivity [1,3,6,7].

Unlike other mental disorders, like anxiety or schizophrenia, where the burden of disease comes almost exclusively from YLDs [18,19], disorders related to the consumption of psychoactive substances make a much greater contribution to premature mortality. This is evident in the consumption of alcohol and opioids/cocaine, according to reports from different studies [7,20], which shifts the burden of the problem towards younger ages, with a greater impact on males [7,8,9,20,21].

Rate heterogeneity by department is notable. Quindío and Nariño surpassed 1600 DALYs per 100,000 inhabitants, while La Guajira, Casanare, and Vaupés reported figures below 132 DALYs. Although part of this variability may be related to differences in diagnostic capacity, healthcare access, and quality of reporting systems [9,22]. The widespread increase in rates across departments suggests a true epidemiological phenomenon rather than solely improvements in information systems.

Departments with higher DALY rates, such as Quindío, Nariño, Norte de Santander, Risaralda, and Antioquia, present heterogeneous but epidemiologically relevant contextual characteristics, including border dynamics, drug-trafficking corridors, socioeconomic inequalities, and differential access to specialized mental health and addiction treatment services. Border territories such as Nariño and Norte de Santander, which share international boundaries with Ecuador and Venezuela, respectively, may experience increased population mobility and illicit trade flows that contribute to greater exposure to psychoactive substance use and associated harms [11,13].

Likewise, a geographic clustering pattern was identified in the Colombian Coffee-Growing Region (Eje Cafetero), where Quindío, Risaralda, Caldas, and Antioquia consistently exhibited elevated DALY rates. In contrast, departments from the Orinoquía and Amazon regions, including Vichada, Arauca, Casanare, Vaupés, and Guaviare, presented the lowest DALY rates, although these findings could partially reflect underdiagnosis, lower reporting capacity, and barriers to healthcare access in geographically dispersed populations [13].

These territorial differences should also be interpreted in light of broader social determinants, including socioeconomic inequalities, youth unemployment, informal employment, and gaps in access to education and healthcare services, which may influence both substance-use patterns and case detection [3,4,13].

With regard to sex, the burden is greater in men, who contribute 73.5% of the DALYs, with rates of 823.50 against 148.40 per 100,000 in women. The mean age is low (31–32 years), with early peaks in cannabis (23 years), intermediate peaks in cocaine and “other drugs”, and later in alcohol (40 years). This pattern coincides with international reports [9,22]. The higher burden observed among men likely reflects both a higher prevalence of substance use and greater exposure to severe outcomes, including injuries, violence, overdose, and other risk-related behaviors. National surveys conducted in Colombia consistently report a higher prevalence of alcohol and psychoactive substance use among men, particularly among adolescents and young adults [13,22,23]. These differences may also be influenced by gender-related behavioral patterns and greater participation in high-risk activities associated with alcohol and psychoactive substance use [2,6].

Differences by substance type also revealed distinct epidemiological patterns. Cannabis-related disorders were associated with younger age groups, whereas alcohol-related burden showed a greater contribution to premature mortality and older age groups. Opioid-related mortality presented lower mean ages, suggesting higher lethality among younger populations. These findings are consistent with international evidence showing heterogeneous health impacts according to substance type, consumption patterns, and associated social contexts [9]. In addition, regional variations in drug-related burden may reflect differences in local drug markets, trafficking routes, and availability of mental health and addiction treatment services [11].

“The increase in YLLs observed during the study period, concentrated among men and occurring predominantly during the fourth and fifth decades of life, is consistent with the elevated risk of injuries, overdose, and comorbidities associated with harmful substance use [7,8].

Globally, alcohol represented 132.60 million DALYs (5.1% of the total burden) in 2016 and around 2 million deaths, while drugs caused 31.80 million DALYs. Overall, alcohol accounted for approximately 76% of the burden attributable to substances [7,9].

In Latin America, the patterns show male predominance and high contribution of alcohol to injuries and non-communicable diseases, with variability among countries according to control policies and access to treatment [9,11]. In Colombia, as in Latin America, the patterns are of male predominance and high contribution of alcohol to the burden of disease, although their crude rates are higher than those reported in countries like Chile, Mexico, and Spain.

In Chile, mental, neurological and substance use disorders constitute a quarter of the DALYs [23], which is similar to the impact of alcohol and drugs in Colombia, although with a greater weight of chronic morbidity [24]. In Mexico, the results of the 2019 GBD study estimated an important contribution of disorders related to substance consumption to the national burden, with interstate heterogeneity and male predominance [25] as found in this study for Colombia. In Europe, alcohol consumption is one of the main risk factors of mortality and DALY, principally due to traffic injuries; nonetheless, age-adjusted rates are lower than in several Latin American countries due to better control of comorbidities [26,27,28].

In Colombia, the ENCSPA 2019 reported a prevalence of alcohol consumption in the last month of 30.1%, with departmental and social variations [13]. In the school-age population (12–17 years), close to 50% had at some time consumed alcohol and 31% did so during the last month, with an average age of onset of 13–14 years [22]. In Bogotá, the current prevalence of alcohol consumption went from 36.5% in 2016 to 39.0% in 2022, while once-in-a-lifetime marijuana use increased from 13.4% to 14.9% [27]. In Antioquia, the 2021 study showed that 68.9% of those consulted had consumed alcohol during the last year, with an average age of onset of 15 years [24]. These data reflect a sustained consumption trend, particularly among the youth.

Taken together, these findings reinforce the need for territorially differentiated public health strategies, including population-based measures, selective prevention programs, and continuous therapeutic care to reduce the burden associated with alcohol and psychoactive substance use.

## 5. Conclusions

This study evidences that consumption of alcohol and other psychoactive substances constitutes a significant burden for public health in Colombia. The period from 2016 to 2022 reflects a growing impact, with an annual average increase of 3261 DALYs, with prevalence in young men and with heterogeneous rates among departments, pointing to both epidemiological factors and possible variations in the detection and response capacity of health systems.

Alcohol and drug consumption in Colombia is configured as a central determinant of health loss, which demands multisectoral and sustained responses. Implementation of evidence-based policies incorporating gender, territorial, and life-course approaches is fundamental to mitigating the impact of alcohol and psychoactive substance use on premature mortality and loss of healthy life years.

### Limitations

This study has several limitations inherent to the use of secondary administrative health databases. First, morbidity estimates may be affected by differential access to healthcare services, as the RIPS database only includes individuals who sought care within the healthcare system. Second, mortality estimates may be influenced by errors in death certification and potential misclassification of the underlying cause of death. To reduce these limitations, mortality records from both healthcare institutions and forensic medicine systems were included, allowing incorporation of deaths occurring both within and outside healthcare facilities. Additionally, the classification of mortality followed the WHO burden-of-disease methodological approach based on the underlying cause of death. Nevertheless, underreporting and diagnostic misclassification may persist, particularly in regions with limited healthcare access or lower reporting capacity.

Another important limitation is that the study estimated the direct burden associated with alcohol- and substance-use disorders identified through ICD-10 diagnostic codes, rather than the full attributable burden of all diseases causally linked to substance use.

Finally, international comparisons should be interpreted with caution, given that crude rates are reported here, whereas global studies generally publish age-adjusted rates.

## Figures and Tables

**Table 1:** DALY rates associated with alcohol and psychoactive substance use by department in Colombia, 2016–2022 and cumulative period.

Department	2016	2022	Period
Amazonas	2.5 (0.3–4.6)	63.6 (63.6–257)	498.1 (342.8–653.3)
Antioquia	75.7 (66.1–85.2)	116.6 (116.6–139.1)	889.2 (858.4–920)
Arauca	31.3 (0–84.5)	0 (0–86.6)	177.5 (97.1–258)
Archipiélago de San Andrés	0.2 (0–0)	16.9 (16.9–27.7)	153.1 (50.4–255.8)
Atlántico	6.9 (6.5–7.4)	36.6 (36.6–58.9)	247.5 (222.3–272.6)
Bogotá, D.C.	23.5 (21.1–25.9)	98 (98–116.8)	443.3 (422.9–463.7)
Bolívar	15.2 (8.9–21.4)	23.8 (23.8–35.2)	244.4 (216.8–272.1)
Boyacá	13.1 (3.7–22.5)	30.9 (30.9–63.8)	215 (180.2–249.7)
Caldas	55.1 (29.2–81.1)	232.4 (232.4–303.5)	770.9 (710.4–831.3)
Caquetá	6.8 (5.6–8)	31.5 (31.5–37.4)	223.4 (173–273.8)
Casanare	7.6 (6.4–8.9)	9.5 (9.5–12.3)	83.4 (61.3–105.4)
Cauca	14 (8.8–19.1)	27.4 (27.4–60.9)	350.7 (292.2–409.2)
Cesar	15.4 (5.9–25)	54.3 (54.3–105.1)	284.3 (237.8–330.8)
Chocó	9 (0–21.6)	10.3 (10.3–92)	132.8 (73.2–192.3)
Córdoba	5.9 (4–7.7)	30.2 (30.2–52.5)	201.5 (183.2–219.8)
Cundinamarca	11.8 (11.3–12.4)	19.8 (19.8–28.2)	138.1 (129.2–147.1)
Guainía	1.6 (0–0)	0 (0–407)	274.8 (0–562)
Guaviare	34 (0–118.3)	13.8 (13.8–21.7)	223.8 (64.5–383)
Huila	8.9 (8–9.7)	30.9 (30.9–37.5)	273.7 (241.7–305.7)
La Guajira	20.1 (0–44.2)	10.4 (10.4–28.1)	131.8 (94–169.5)
Magdalena	3 (2.6–3.4)	11.6 (11.6–30.2)	140.4 (109.2–171.7)
Meta	9.1 (0–22.3)	36.6 (36.6–81.2)	136.6 (107.6–165.7)
Nariño	44.7 (36.2–53.2)	37.3 (37.3–79.4)	1624.3 (1539.5–1709)
Norte de Santander	24.6 (5.6–43.6)	84.8 (84.8–111)	1008 (953.2–1062.7)
Putumayo	16.4 (14.4–18.4)	4.9 (4.9–74.7)	265.1 (213.7–316.5)
Quindío	155 (116.6–193.5)	210.6 (210.6–311.8)	1779.5 (1681.3–1877.7)
Risaralda	75.9 (43.3–108.4)	132.3 (132.3–206.1)	909.5 (825.6–993.4)
Santander	35.6 (22.4–48.8)	47.7 (47.7–71.6)	336.8 (305.6–368)
Sucre	10 (9.2–10.8)	73.7 (73.7–116.6)	322.1 (280.9–363.4)
Tolima	9.4 (1.5–17.4)	24.5 (24.5–61.8)	220.9 (183.9–257.9)
Valle del Cauca	35.2 (25.7–44.7)	50.9 (50.9–81.1)	426.9 (389.6–464.3)
Vaupés	0.3 (0–0)	4.5 (4.5–12)	27.5 (19.9–35)
Vichada	0.5 (0–0)	1.6 (1.6–4.9)	164.6 (0–330.6)
Colombia	30.2 (28–32.4)	74.3 (74.3–80.9)	478.1 (469.7–486.5)

## Data Availability

The morbidity and mortality data analyzed in this study were obtained from information systems managed by the (https://www.minsalud.gov.co/Portada/index.html), (accessed on 5 September 2025). The data was retrieved through the SISPRO (https://www.sispro.gov.co/Pages/Home.aspx), (accessed on 5 September 2025), a national platform that consolidates multiple health information systems, including RUAF and RIPS. Access to these databases is restricted and requires a formal request to the MSPS. This process is conducted in accordance with Statutory Law 1581 of 2012 [29], which permits the use of information for historical, statistical, and scientific purposes. Researchers seeking access must request authorization and credentials by contacting the MSPS at (sispro_bodega@minsalud.gov.co). Once the request is approved, access to the SISPRO server is granted, allowing data to be queried and extracted using analytical tools based on SQL Server Analysis Services, typically through its integration with Microsoft Excel. For the purposes of this study, mortality records were obtained from the RUAF—Non-Fetal Mortality database, while morbidity data were derived from the RIPS. Demographic data used in the analyses correspond to the official population projections for the period 2016–2022, published by the DANE (https://www.dane.gov.co/index.php), (accessed on 5 September 2025).

## Supplementary Materials

The following supporting information can be downloaded at: https://www.mdpi.com/article/10.3390/ijerph23050659/s1, Table S1: Data downloaded from SISPRO. Table S2: GATHER table. Table S3. Morbidity and mortality cases related to alcohol and psychoactive substance use by year, substance, sex, ICD-10 diagnosis, and department in Colombia, 2016–2022. Table S4. Years lived with disability (YLD), years of life lost (YLL), and disability-adjusted life year (DALY) rates according to sex and department in Colombia, 2016–2022.

## Abbreviations

CIOMS	Council for International Organizations of Medical Sciences
DALYs	Disability-Adjusted Life Years
DANE	National Administrative Department of Statistics
GBD	Global Burden of Disease
GHE	Global Health Estimates
RIPS	Individual Health Service Provision Records
RUAF	Single Affiliation Registry
YLDs	Years lived with disability
YLLs	Years of Life Lost
SISPRO	Integrated Social Protection Information System
MSPS	Ministry of Health and Social Protection
ENCSPA	National Survey on Psychoactive Substance Consumption

## References

[1] Zhang T., Sun L., Yin X., Chen H., Yang L., Yang X. (2024). Burden of drug use disorders in the United States from 1990 to 2021 and its projection until 2035: Results from the GBD study. BMC Public Health.

[2] GBD 2016 Alcohol and Drug Use Collaborators (2018). The global burden of disease attributable to alcohol and drug use in 195 countries and territories, 1990–2016: A systematic analysis for the Global Burden of Disease Study 2016. Lancet Psychiatry.

[3] Danpanichkul P., Duangsonk K., Díaz L.A., Chen V.L., Rangan P., Sukphutanan B., Dutta P., Wanichthanaolan O., Ramadoss V., Sim B. (2025). The burden of alcohol and substance use disorders in adolescents and young adults. Drug Alcohol Depend..

[4] Piedra R., Masa R., Chamba T., Ruiz S. (2020). El consumo de sustancias psicoactivas y su influencia en el desarrollo integral. J. Bus. Entrep. Stud..

[5] Bardach A.E., Alcaraz A.O., Ciapponi A., Garay O.U., Riviere A.P., Palacios A., Cremonte M., Augustovski F. (2019). Alcohol consumption’s attributable disease burden and cost-effectiveness of targeted public health interventions: A systematic review of mathematical models. BMC Public Health.

[6] Alpert H.R., Slater M.E., Yoon Y.-H., Chen C.M., Winstanley N., Esser M.B. (2022). Alcohol Consumption and 15 Causes of Fatal Injuries: A Systematic Review and Meta-Analysis. Am. J. Prev. Med..

[7] GBD 2016 Alcohol Collaborators (2018). Alcohol use and burden for 195 countries and territories, 1990–2016: A systematic analysis for the Global Burden of Disease Study 2016. Lancet.

[8] World Health Organization (2018). Global Status Report on Alcohol and Health 2018.

[9] Li J., Li X., Shen Y., Yang X., Liu T. (2025). Global burden attributed to alcohol and drug use among adolescents and young adults, 1990–2019: Results from the Global Burden of Disease Study 2019. BMJ Open.

[10] Institute for Health Metrics and Evaluation (IHME) (2020). Global Burden of Disease Study 2019 (GBD 2019) Results.

[11] Castaldelli-Maia J., Wang Y.-P., Brunoni A.R., Faro A., Guimarães R.A., Lucchetti G., Martorell M., Moreira R.S., Pacheco-Barrios K., Rodriguez J.A.B. (2023). Burden of disease due to amphetamines, cannabis, cocaine, and opioid use disorders in South America, 1990-2019: A systematic analysis of the Global Burden of Disease Study 2019. Lancet.

[12] Ministry of Justice, Law—Drug Observatory of Colombia, National Forensics Institute (2022). Mortality Study Associated with the Consumption of Psychoactive Substances 2013–2020.

[13] Ministerio de Justicia y del Derecho, Observatorio de Drogas de Colombia, Oficina de las Naciones Unidas Contra la Droga y el Delito (2020). Boletín Técnico—Encuesta Nacional de Consumo de Sustancias Psicoactivas (ENCSPA) 2019.

[14] Department of Data and Analytics Division of Data Analytics and Delivery for Impact WHO (2024). WHO Methods and Data Sources for Global Burden of Disease Estimates 2000–2021.

[15] World Health Organization (2020). WHO Methods and Data Sources for Global Burden of Disease Estimates 2000–2019.

[16] Ministerio de Salud de Colombia (1993). Resolución 8430 de 1993, Por la Cual se Establecen las Normas Científicas, Técnicas y Administrativas Para la Investigación en Salud.

[17] Council for International Organizations of Medical Sciences (CIOMS) (2016). International Ethical Guidelines for Health-Related Research Involving Humans.

[18] GBD 2019 Mental Disorders Collaborators (2022). Global, regional, and national burden of 12 mental disorders in 204 countries and territories, 1990–2019: A systematic analysis for the Global Burden of Disease Study 2019. Lancet Psychiatry.

[19] Quintero Díaz K.J., Gutierrez Lesmes O.A., Salamanca Ramos E. (2025). Burden of Mental and Behavioral Disorders in Colombia, 2022: A Subnational Analysis Based on Disability-Adjusted Life Years. Int. J. Environ. Res. Public Health.

[20] Moan I.S., Bye E.K., Rossow I. (2021). Stronger alcohol-violence association when adolescents drink less? Evidence from three Nordic countries. Eur. J. Public Health.

[21] Sampaio G., Lima G., de Souza S., Soares D. (2024). Use of psychoactive substances among university students from 2019 to 2020: A systematic review. Brain Behav. Immun.-Health.

[22] Observatorio de Drogas de Colombia, Ministerio de Justicia y del Derecho (2016). Estudio Nacional de Consumo de Sustancias Psicoactivas en Población Escolar, Colombia 2016.

[23] Beroíza-Valenzuela F. (2024). The challenges of mental health in Chilean university students. Front. Public Health.

[24] Gobernación de Antioquia, Secretaría Seccional de Salud y Protección Social de Antioquia, Escuela Contra la Drogadicción, ASCODES SAS (2022). Estudio de Consumo de Sustancias Psicoactivas para el Departamento de Antioquia, sus Subregiones y Medellín—2021.

[25] García-Pacheco J.Á., Ortega M.d.L.T., Borges G. (2024). The burden of mental disorders in Mexico, 1990-2019: Mental and neurological disorders, substance use, suicides, and related somatic disorders. Span. J. Psychiatry Ment. Health.

[26] Murray C.J.L., Aravkin A.Y., Zheng P., Abbafati C., Abbas K.M., Abbasi-Kangevari M., Abd-Allah F., Abdelalim A., Abdollahi M., Abdollahpour I. (2020). Global burden of 87 risk factors in 204 countries and territories, 1990–2019: A systematic analysis for the Global Burden of Disease Study 2019. Lancet.

[27] Secretaría Distrital de Salud de Bogotá, Observatorio de Salud de Bogotá—SaluData (2022). Prevalencia Consumo Actual de Bebidas Alcohólicas, Tabaco, Sustancias Ilícitas en Bogotá, D.C., 2016–2022. https://saludata.saludcapital.gov.co/osb/indicadores/prevalencia-consumo-actual/.

[28] Borges G., García-Pacheco J.Á., Familiar-Lopez I. (2021). Global estimates of the attributable risk of alcohol consumption on road injuries. Alcohol. Clin. Exp. Res..

[29] Congreso de Colombia Ley 1581 de 2012—Ley de Protección de Datos Personales 2012. https://www.funcionpublica.gov.co/eva/gestornormativo/norma_pdf.php?i=49981.

