# Supplementary material for: Burden of Disease Due to Consumption of Alcohol and Other Drugs in Colombia, 2016–2022: A Subnational Regional Analysis
Source: Int J Environ Res Public Health. 2026 May 15;23(5):659. doi: 10.3390/ijerph23050659 (PMC13206594; doi:10.3390/ijerph23050659)
Supplement: Supplementary file 1 [file ijerph-23-00659-s001.zip › Supplementary Materials Table S2.pdf]

GATHER table

	Item	Comment for compliance.
1	Define the indicator(s), the population (including age, sex, and geographical data), and the period(s) for which the estimates were produced.	The study used Disability-Adjusted Life Years (DALYs) as the main indicator, integrating Years Lived with Disability (YLDs) and Years of Life Lost (YLLs), following the World Health Organization's methodological guidelines under the Global Health Estimates 2024 framework.<br><br>The study population included all residents of Colombia during the period 2016–2022, without age restrictions, disaggregated by sex, age groups, and department of residence, covering all 32 departmental territorial entities of the country.<br><br>All officially registered cases and deaths in the national health information systems during the study period were included, provided they had a diagnosis within the group of alcohol and other drug use according to ICD-10.
2	State the funding sources for the activity.	This research was funded by the Ministry of Science, Technology, and Innovation of Colombia (MINCIENCIAS) and the University of Los Llanos, through the project titled: *"Development of a Mental Health, Family, and Social Coexistence Observatory for the design of comprehensive strategies, knowledge management, and the formulation of policies, plans, and programs in the Colombian Orinoquía region"* (Code 112291891873, Contract 655 of 2022).<br><br>The funders had no role in the study design, data collection, data analysis, interpretation of results, or the decision to publish the findings.
3	Describe how the data were identified and obtained.	Secondary official sources from the Integrated Social Protection Information System (SISPRO) were used. Morbidity data were obtained from the Individual Registry of Health Service Provision (RIPS), which consolidates medical care records reported by Health Service Provider Institutions (IPS) and validated by Benefit Plan Administration Entities and the Ministry of Health and Social Protection (MSPS) for the period 2016–2022. Cases were identified based on the recorded primary diagnosis.<br><br>Mortality data were extracted from the Unique Affiliation Registry (RUAF), a component of the Vital Statistics System, using the underlying cause of death reported by the MSPS and coded according to ICD-10 by the National Administrative Department of Statistics (DANE).<br><br>Access to these databases is restricted and regulated under national data protection regulations. Population denominators for rate calculations were obtained from official projections by department, year, and sex published by DANE for 2016–2022.
4	Specify inclusion and exclusion criteria, and all specific exclusions.	All morbidity and mortality records for the period 2016–2022 with a diagnosis classified within the group of alcohol and other psychoactive substance use, according to ICD-10, and registered in Colombia's official health information system databases were included.<br><br>Cases coded within ICD-10 ranges F19.0–F19.9, X42.0–X42.9, and X45.0–X45.9, corresponding to multiple substance use, accidental poisoning by narcotics and psychodysleptics, and accidental alcohol poisoning, respectively, were redistributed

	Item	Comment for compliance.
5		proportionally by age group, sex, and department to assign them to the specific substances analyzed in the study.<br><br>Records with incomplete information, inconsistencies in key variables (age, sex, or department), missing primary diagnosis or underlying cause of death, and duplicate entries were excluded
5	Provide information on all data sources and their main characteristics. For each source used, include reference information or institutional contact, the represented population, data collection method, year(s) of data collection, sex and age range, diagnostic criteria or measurement method, and sample size, if applicable.	Mortality data were obtained from the non-fetal deaths database of the RUAF, a component of the Colombian Vital Statistics System, available through SISPRO. This database has national coverage and includes death certificates completed by medical personnel and coded according to ICD-10 by DANE. The underlying cause of death was analyzed for the period 2016–2022, covering both sexes and all ages.<br><br>Morbidity data were obtained from RIPS, a national administrative database that consolidates health care records reported by Health Service Provider Institutions and validated by health authorities. The primary diagnosis or, in the case of injuries, the external cause was considered, following ICD-10 codes, for the period 2016–2022.<br><br>Both databases include variables on sex, age, department, and diagnostic code. The full set of available records was used.
6	Mention and describe all types of input data that may be subject to substantial bias	The databases used may be affected by health care access bias, since RIPS only includes individuals who sought care within the health system, potentially underestimating the true morbidity burden. Mortality may be influenced by competing risk bias, where deaths are attributed to causes other than the underlying substance use disorder, as well as by errors in the certification and coding of the underlying cause of death.<br><br>To mitigate these biases, mortality estimates were based on the underlying cause of death, following the World Health Organization burden-of-disease methodology, and included all deaths registered in both the hospital network and legal medicine records, improving coverage of out-of-hospital events.<br><br>Potential biases from underreporting and diagnostic misclassification remain, as these could not be corrected due to reliance on secondary administrative data sources.
7	List any other input data and specify their source.	To estimate the rates, the population denominators were obtained from the official population projections produced by the National Administrative Department of Statistics (DANE) of Colombia, available at: https://www.dane.gov.co/index.php/estadisticas-por-tema/demografia-y-poblacion/proyecciones-de-poblacion
8	Provide all input data in a file from which data can be efficiently extracted (e.g., preferably in a spreadsheet rather than a PDF), including all relevant metadata specified in item 5. For data that cannot be publicly shared for ethical or legal reasons, such as third-party data, provide the name of the	The databases used in this study are provided as supplementary material.

	Item	Comment for compliance.
	contact person or institution holding the rights.	
9	Provide a conceptual overview of the data analysis method; a diagram may be helpful	The analysis was based on the DALY metric developed within the GBD framework, which integrates into a single time-based unit both premature mortality and non-fatal morbidity attributable to alcohol and other psychoactive substance use.<br><br>DALYs were estimated as the sum of YLL and YLD:<br><br>DALY = YLL + YLD<br><br>This approach allows for comparisons of burden across different causes and territories by using time as a standard measurement unit.
10	Describe in detail all stages of data analysis, including the mathematical formulas used. This description should include, as applicable, data cleaning, preprocessing, adjustments, weighting of data sources, and the mathematical or statistical models applied.	Morbidity and mortality data were extracted as pivot tables.<br><br>Subsequently, the datasets were converted into individual-level databases using Power Query (Microsoft Excel) and Power BI desktop. This process included the disaggregation of grouped cases and the assignment of categorical variables (sex, age, department, primary diagnosis, or underlying cause of death) to each individual record.<br><br>A data cleaning process was conducted, which included the removal of duplicate records, cases without a primary diagnosis or without an underlying cause of death, and records with inconsistencies in key variables.<br><br>Cases coded as multiple substance use or unspecified poisonings (F19.0–F19.9, X42.0–X42.9, and X45.0–X45.9) were proportionally distributed according to age group, sex, and department for their assignment to specific substances.<br><br>No sampling weights were applied, as the full set of available records was used.<br><br>The burden of disease was estimated using the GBD methodology, following the structure:<br><br>DALY = YLL + YLD<br><br>Data processing and statistical analysis were performed using SPSS software.
11	Describe how the different models were evaluated and how the model(s) used were selected.	The study applied the abbreviated methodology proposed by the World Health Organization (WHO) for estimating the Global Burden of Disease (GBD). This approach was selected based on its standardized analytical framework, its internal methodological consistency, and its widespread acceptance in international burden-of-disease reporting.<br><br>Rather than comparing alternative statistical models, the selection was guided by conceptual and methodological criteria. The WHO abbreviated framework ensures comparability across causes, populations, and time periods, and aligns with established global reporting standards. Its structured and transparent calculation of Years of Life Lost (YLL), Years Lived

	Item	Comment for compliance.
		with Disability (YLD), and Disability-Adjusted Life Years (DALYs) makes it particularly suitable for analyses based on administrative health data.<br><br>Accordingly, the methodology was chosen for its robustness, reproducibility, and coherence with international burden-of-disease estimation practices.
12	Report the results of model performance evaluation, if conducted, and the corresponding sensitivity analyses	Not applicable.
13	Describe the methods used to calculate uncertainty in the estimates. Indicate which sources of uncertainty were considered in the analysis and which were not.	Uncertainty in the estimates was assessed using the bootstrap method implemented in SPSS. This statistical resampling technique was applied to obtain empirical distributions of the estimates and their confidence intervals. The procedure follows the standard approach based on the standard error of the sum and the Student’s t distribution.<br><br>1. Calculation of the sum<br>For a variable X_i with n observations:<br>Sum=∑i=1nXi\text{Sum} = \sum_{i=1}^{n} X_iSum=i=1∑nXi<br><br>2. Calculation of the standard error of the sum<br>SPSS first calculates the sample standard deviation (s) and then derives the standard error of the sum from the standard error of the mean, as follows:<br>SEX¯=snSE_{\bar{X}} = \frac{s}{\sqrt{n}}SEX¯=ns SEsum=SEX¯×n=s×nSE_{\text{sum}} = SE_{\bar{X}} \times n = s \times \sqrt{n}SEsum=SEX¯×n=s×n<br><br>3. Calculation of the confidence interval<br>SPSS uses the Student’s t distribution with n – 1 degrees of freedom (for small samples) or the normal distribution (when n is large), and the confidence level specified (by default, 95%).<br>UI=Sum±tα/2, n−1×SEsumaUI = \text{Sum} \pm t_{\alpha/2,\,n-1} \times SE_{\text{sum}}IC=Sum±tα/2,n−1×SEsum
14	Indicate how to access the analytical or statistical source code used to produce the estimates.	Not applicable.
15	Provide the published estimates in a file format that allows efficient data extraction	The estimates are presented in a complementary annex in .xlsx (Excel) format, which enables efficient data extraction and reuse. This file is available as a supplementary annex.
16	Provide a quantitative measure of the uncertainty of the estimates (e.g., confidence intervals).	Uncertainty intervals were generated for the indicator rates.
17	Interpret the results in light of the available evidence. If this is an update of previous estimates, describe the reasons for any changes	At the subnational level, available evidence primarily derives from the National Survey on Psychoactive Substance Use, which reports prevalence estimates by department. However, these data do not provide comprehensive burden-of-disease estimates, limiting the ability

	Item	Comment for compliance.
18		to fully assess the differential impact of alcohol and other psychoactive substance use across territories or to prioritize interventions according to their relative magnitude.<br><br>Within this context, the present study represents a novel and recent effort to estimate the burden attributable to alcohol and psychoactive substance use at the departmental level in Colombia. Rather than constituting a direct update of previous estimates, it offers a complementary DALY-based assessment that enhances understanding of the health impact and provides robust technical evidence to inform public health planning and promote a more equitable allocation of resources.
18	Explain the limitations of the estimates. Present an analysis of the model assumptions and the data limitations that affect the interpretation of the estimates.	This study is based on secondary administrative records and is therefore subject to potential underreporting and diagnostic misclassification. The validity of the estimates depends on the accuracy and completeness of national health information systems, which may introduce variability in the identification and recording of cases related to alcohol and psychoactive substance use.<br><br>Although forensic medicine data were incorporated to reduce bias in the estimation of attributable mortality, information gaps persist—particularly in remote or underserved areas where limitations in death registration coverage and data quality remain. These constraints may affect the estimation of the Years of Life Lost (YLL) component.<br><br>Finally, comparisons with international studies should be interpreted with caution. The present analysis reports crude rates, whereas global burden of disease studies typically present age-standardized rates. This methodological difference may influence the relative magnitude of the estimates and limit their direct external comparability
